# Envelope 2 protein phosphorylation sites S75 & 277 of hepatitis C virus genotype 1a and interferon resistance: A sequence alignment approach

**DOI:** 10.1186/1743-422X-8-71

**Published:** 2011-02-15

**Authors:** Samia Afzal, Muhammad Idrees, Muhammad Ali, Muhammad Ilyas, Abrar Hussain, Madiha Akram, Sadia Butt, Sana Saleem, Irshad ur Rehman, Liaqat Ali, Muhammad Shahid

**Affiliations:** 1 National Centre of Excellence in Molecular Biology, 87-West Canal Bank Road, Thokar Niaz Baig, Lahore-53700, University of the Punjab, Lahore, Pakistan

## Abstract

**Background:**

Hepatitis C is a major health problem affecting more than 200 million individuals in world including Pakistan. Current treatment regimen consisting of interferon alpha and ribavirin does not always succeed to eliminate virus completely from the patient's body.

**Results:**

Interferon induced antiviral protein kinase R (PKR) has a role in the hepatitis C virus (HCV) treatment as dsRNA activated PKR has the capacity to phosphorylate the serine and threonine of E2 protein and dimerization viral RNA. E2 gene of hepatitis C virus (HCV) genotype 1 has an active role in IFN resistance. E2 protein inhibits and terminates the kinase activity of PKR by blocking it in protein synthesis and cell growth. This brings forward a possible relation of E2 and PKR through a mechanism via which HCV evades the antiviral effect of IFN.

**Conclusion:**

A hybrid in-silico and wet laboratory approach of motif prediction, evolutionary and structural anlysis has pointed out serine 75 and 277 of the HCV E2 gene as a promising candidate for the serine phosphorylation. It is proposed that serine phosphorylation of HCV E2 gene has a significant role in interferon resistance.

## Background

Hepatitis C virus is a key universal health issue [1] affecting approximately 200 million individuals worldwide and over 4 million in the United States alone, where it is the most common blood-borne infection [2]. It is the main reason of persistent liver infection and the most familiar sign for liver transplantation [3]. In 60-85% cases HCV develops to cirrhosis and hepatocellular carcinoma [4]. Presently, in Pakistani population 17 million people are infected with HCV and 8-10% individuals are HCV carriers [5]. HCV is a member of genus, hepacivirus, family Flaviviridae and is a positive sense single stranded RNA virus [6]. HCV genome is about 9.6 kb in length [7]. The large open reading frame of Viral RNA having 5` and 3` untranslated regions which is translated into a single polypeptide of 3010 to 3033 amino acids. Which is processed by host as well as viral proteases to yield 10 mature individual proteins out of which 3 are structural and 7 are nonstructural [8,9]. Detailed structure of HCV virus is still unclear. However, the infectious viral particles are composed of lipid envelope glycoproteins E1 and E2 [10].

In spite of much recent advancements, still no vaccine is available against HCV infection. The current therapy for HCV infection is pegylated interferon alpha separately or in combination with ribavirin [11] but it eradicates the virus in only 50-80% of cases and has serious side effects [12]. IFN system is the first line of protection against viral infection in mammals [13]. Many viruses have developed mechanisms to dodge the IFN-dependent cellular response [14]. Amongst these HCV is a significant example in which (70-80%), cases runs away the host defenses and develops a chronic infection. Pathological outcomes of HCV infection changes from individual to individual, unstable from asymptomatic state to liver fibrosis, steatosis, finally to hepatocellular carcinoma [15,16]. The factors upon which the success or failure of the antiviral therapy depends are unspoken yet, and their recognition characterizes a main confront in HCV virology [11]. IFN-interacts with cells and modifies the expression of a number of genes [17,18]. E2 glycoprotein is the first viral factor that gets in touch with the host cell surface receptors thus it has an important role in vaccine designing and drug objective [19,20]. Primary objective of HCV vaccine is to initiate potent humoral responses against E2 protein [21].

After the death of infected cells, virus particles are discharged and they can infect the cells present in close proximity. Interferon is released from the infected cells to inform the neighboring cells about the presence of virus. In its rapid response, PKR is produced by these adjoining cells. HCV resistance to IFN-treatment is partially related to inhibition of interferon induced anti-viral protein PKR. Interferon induce many protective mechanisms in cells and amongst these the major role in cell protection from many viruses is illustrated by double-stranded RNA (dsRNA)-activated protein kinase PKR [22,23].

Two HCV proteins (NS5A and E2) are involved in IFN resistance through inhibition of the IFN-α induced double stranded-RNA (dsRNA)-activated protein kinase (PKR) [24,25]. PKR is a kinase enzyme that reveals varied activities. PKR exhibits autophosphorylation of many serine and threonine positions and dimerization of dsRNA. It also phosphorylates the translation initiation factor eIF-2 (α subunit) that directs towards blockage of protein synthesis [26]. Because of these properties, PKR is taken as an arbitrator of antiviral and anti-inflammatory role of IFN-α [27]. E2 protein blocks PKR activation to bypass its function [24-28]. Taylor *et al.*, (1999) [25] reported that HCV E2 protein encloses a 12 amino acid sequence domain that is highly homologous to autophosphorylation positions of PKR and initiation factor eIF2. PKR and eIF2 form a phosphorylation homology domain (PePHD). E2 protein inhibits kinase activity of PKR and terminates its blocking role in protein synthesis and cell growth. This suggests that the relationship of E2 and PKR can be considered as a major mechanism by which HCV evades the antiviral effect of IFN.

## Methods

### HCV RNA Detection and identification of E2 gene

Serum samples were collected at the Division of Molecular Virology, National Centre of Excellence in Molecular Biology, University of the Punjab, Lahore. HCV 1a genotype serum samples were used in the current study. 100 μl serum was used to extract viral RNA by using RNA Isolation Kit (Sacace, Italy). Primer 3 software was used to designed primer for the amplification of E2 gene. At 5' end a start codon was introduced artificially in the primers for expression of gene in mammalian cell culture. The required amplified product was of approximately 1089 bps which was confirmed on ethidium bromide stained 1.2% agarose gel evaluated under UV transilluminator and photographed. The necessary band was cut out from the gel and the DNA was extracted with DNA isolation kit (Fermentas Inc. Germany). Isolated DNA was suspended in depc treated water and used for further studies.

### Construction of expression vector

Mammalian expression vector pcDNA 3.1 (Invitrogen Tech USA) was used to clone the amplified product encoding E2 gene between Hindi III and EcoR I sites. The plasmid was transformed in chemically-competent cells. The entire ligation reaction was added to a 100 μl aliquot of TOP10F cells. Then 500 μl of SOC medium was added to the cells and incubated at 37°C for 1 hour. Competent cells having plasmid were then selected by spreading the culture onto a Luria-Bertani (LB) agar plate containing 100 μg/ml ampicillin and 12.4 ug/ml tetracyclin. Colonies selection was made by incubating the plate overnight at 37°C. To identify bacteria harboring cloned E2 gene, individual colonies were used to directly inoculate PCR reactions. The PCR reactions were prepared with 5 pmol each of vector-specific primers i.e. T7 (TAATACGACTCACTATAGGG) and BGH (TAGAAGGCACAGTCGAGG). Each reaction was prepared to a final volume of 20 μl. Following amplification, the amplified product was checked on a 1.2%, ethidium bromide stained agarose gel, a successful cloning reaction being visualized as a product at approximately 1.3 kb. Colonies identified as possessing a desired clone were then used to inoculate 3 ml LB culture containing 100 μg/ml ampicillin, shaking at 225 rpm overnight at 37°C. Plasmid was purified using a Plasmid Miniprep Kit, (Fermentas Life Sciences technologies, USA) according to manufacturer's protocol. Quantification of the plasmid prep. was performed by using spectrophotometer. Successful cloning was confirmed through PCR, restriction digestion of plasmid (pcDNA 3.1/myc vector) with EcoR1 & Hindi III and sequencing reaction. At least three clones were sequenced in both directions according to the sequencing protocol.

### Sequence analysis

Applied biosystems prism dye termination method was used to sequence the DNA (PCR products and plasmids) with specific sense and anti sense primers. The sequences reported in this paper have been deposited in genbank database (Accession no. GU736411). In a 10 μl, of total reaction volume, about 300 ng of plasmid, 10 pmol of primer, 1 μl of big dye and 1 μl of dilution buffer were used for each sequencing reaction. Sequencing was achieved in a thermal cycler with parameters; 94°C for 20 s, 58°C for 20 s, 60°C for 4 min, repeated 25 times. The pellet was air dried and DNA was analyzed using an ABI prism sequencer.

### Identification of Phosphorylation sites

To study the possible outcome of predication with help of NetPhos 2.0 software as described by Blom *et al. *(1999) [29]. This software checks the possible post translation modification in the local 1a local strain primary sequence in the amino acid. As a result of analysis the target motif for different kinases is visualized [29,30]. Scansite is kept at low stringency in order to determine the maximum number of sites that may participate in phosphorylation, upon which further predication is done.

### Protein Structure Analysis

An ab-initio model was designed by using I-TASSER as no template model was available in protein Data Bank [31]. Data was uploaded and models were generated. A 3D structure of predicted phosphorylation sites was generated by using two servers, Chimera [31] and SWISS PDB viewer [32] were used to obtain 3D structure. It was checked that potential phosphorylation sites are surface exposed or hidden inside. Five sites were observed at the exposed surface of model (aa: S75, S95, S118, S277, Y211).

### Phylogenetic Analysis

**Figure 1:**
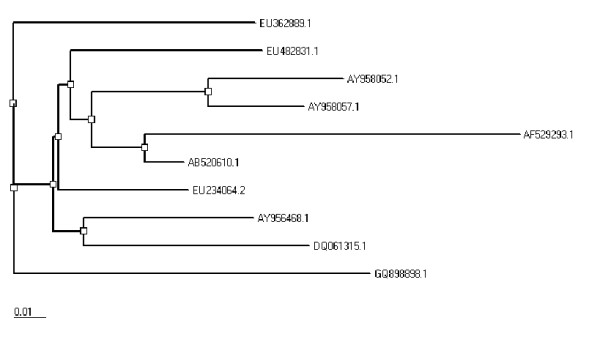
**Phylogeny of local envelope gene sequence with published sequences for HCV genotype 1a from different localities of the world**.

Protein sequences of envelope gene for genotype 1a from different countries of the world (Japan, France, USA, and UK) were obtained from NCBI. All sequences (GQ898898, EU482831, EU234064, EU362889, DQ061315, AY958052, AY958057, AY956468, AB520610, and AF529293) were then aligned with local envelope gene by using CLUSTALW [33]. A neighbor joining tree was generated using PHYLIP (Figure 1) [34].

## Results

To evaluate its function in disease succession, amplified E2 gene was cloned in mammalian expression vector pc DNA 3.1. A CMV promoter is present in pc DNA 3.1 vector that can transduce eukaryotic cells very efficiently for transient and stable expression studies. Clone was sequenced by the applied biosystems prism dye termination method using vector specific and gene specific primers (Accession no. GU736411). Documented sequences for 1a genotype from diversified areas of the world were compared with local envelope gene sequence to get the percentage nucleotide identity (PNI) (Table 1) and CLUSTAL W. Sequence dissimilarities in the envelope genes explained the different levels of disease outcomes in patients infected with same genotype. These nucleotide differences can help out to plan the genotype specific therapy to avoid and minimize HCV infections.

**Table 1:** Comparison of local and published envelope gene sequences.

**Accession No**.	Genotype	Country	% similarity
*GQ898898*	*1a*	*PAKISTAN*	*96%*
*EU482831*	*1a*	*USA*	*92%*
*EU234064*	*1a*	*USA*	*90%*
*EU362889*	*1a*	*USA*	*90%*
*AY956468*	*1a*	*USA*	*91%*
*DQ061315*	*1a*	*USA*	*90%*
*AY958052*	*1a*	*UK*	*91%*
*AY958057*	*1a*	*UK*	*90%*
*AF529293*	*1a*	*FRANCE*	*89%*
*AB520610*	*1a*	*JAPAN*	*88%*

**Figure 2:**
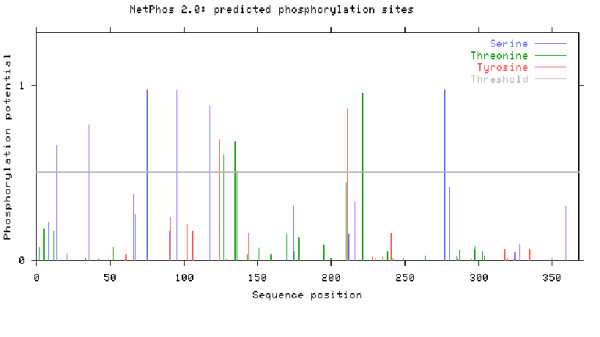
**Phosphorylation sites software calculated in the local HCV envelope gene sequence**.

Possible phosphorylation sites were calculated in the cytoplasmic domain of the E2 protein that can be implicated in the interferon resistance. Twelve putative sites (Figure 2) were chosen as potential phosphorylation sites (S-6, Thr-4, Tyr-2). Stringency of Phosphorylation site predictors was increased as they have tendency to over-predict. Only those motifs were selected that showed a NetPhos score of 0.8 or greater. After it they were also analyzed by Scansite. Finally six sites were predicted by both servers (Table 2).

**Table 2:** Summary of predicted tyrosine phosphorylation sites.

*Phosphorylation sites*						
Site*a	aa*b	Context*c	NetPhos*d	Scansite*e	NetSurfP*f	I-TASSER*g
***75***	S	ERLASCKPL	0.977	Y	E	Yes
***95***	S	YANGSGPEH	0.969	_	E	Yes
***118***	S	VPAQSVCGP	0.885	_	E	Yes
***277***	S	DRDRSELSP	0.978	Y	E	Yes
***221***	T	GPWITPRCL	0.951	Y	E	No
***211***	Y	PEATYSRCG	0.863	Y	B	Yes

a. Phosphorylation sites in the local 1a sequence of the envelope gene.

b. S indicates Serine; T is for Threonine and Y for Tyrosine predictions.

c. Region where the phosphorylation sites are available.

d. Predicted sites by NetPhos with a score of ≥ 0.8. Dashes indicate lack of Phosphorylation sites in that position.

e. Y indicates predicted sites in sequence on "Low Stringency". Dashes indicate lack of Phosphorylation sites in that position.

f. E indicates Exposed sites and B indicates Buried sites. Surface accessibility calculated by NetSurfP.

g. Ab-initio 3D model was constructed by using I-TASSER server.

**Figure 3:**
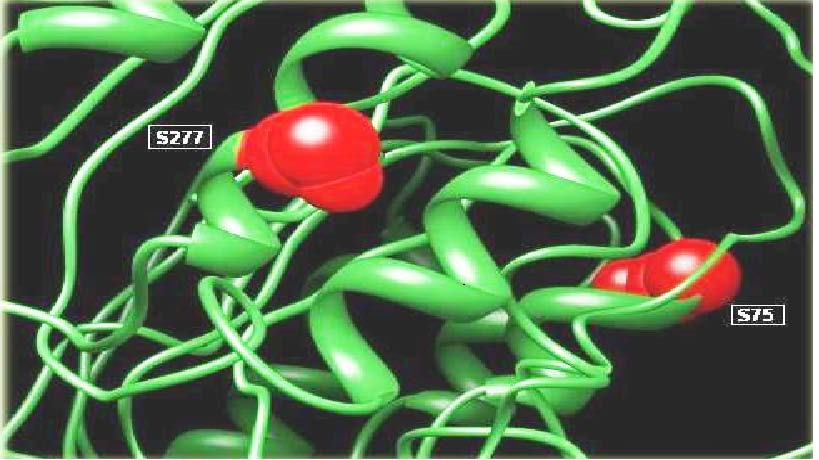
**Visualization of phosphorylation sites S277 and S75 in Tertiary structure of the envelope gene**.

An online server NetSurfP was used to find the surface accessibility of local envelope sequence, as the phosphorylation sites should be exposed on the surface of proteins (Table 2). Discovery Studio and SWISS PDB Viewer were applied to visualize 3D protein structure of the putative sites. PDB structure was built up by using the server I-TASEER (*Ab-initio *protein structure predictor) [35]. After this analysis two phosphorylation sites (S75 and S277) were found to be most reliable sites (Figure 3). Scansite was used for finding the phosphorylation interaction motifs and found that S75 and S277 interact with the CLK2 Kinase and YWHAZ which were then investigated in GeneCards and was confirmed from UniGene and UniProt (Table 3).

**Table 3:** Interacting enzymes predicted by Scansite.

Site*a	Enzyme*b	*Gene Card*	UniGene*c	UniProt*c	*Full Name*
***75***	Clk2 Kinase	CLK2	Yes	P49760	CDC2-like kinase (CLK)
***277***	pST_bind	YWHAZ	Yes	P63104	Tyrosine 3-monooxygenase/tryptophan 5-monooxygenase activation protein, zeta polypeptide
***---***	Clk2 Kinase	CLK2	Yes	P49760	CDC2-like kinase (CLK)

a. Phosphorylation sites in the envelope gene.

b. Observed enzymes by the Scansite.

c. Gene's availability on UniGene and UniProt.

## Discussion

IFN usually causes the transcription of some antiviral genes. However, detailed principal mechanisms exploring high IFN-α resistance in HCV genotype 1a infected patients are still ambiguous. A double stranded RNA activated PKR phosphorylates the translation initiation factor (eLF2) and thus inhibits its protein synthesis ability. A straight relationship was found in a study of E2 protein of HCV genotype 1a and IFN inhibition pathway. It was shown that it inhibits PKR through a 12 amino acid sequence E2-PePHD domain, and for PKR this domain can perform the function of pseudo-substrate. The functions of PKR *i.e. *inhibition of viral protein synthesis and its kinase activity, are blocked by this domain. PKR autophosphorylation sites are highly identical to the PePHD domain in HCV genotype 1a than other genotypes [36].

It has been demonstrated that E2 protein of HCV genotype 1 shares homology with PKR and eIF2 phosphorylation sites and therefore inhibits PKR by binding to it *in vitro *in mammalian and yeast cells. HCV therefore has evolved a mechanism in the form of E2-PKR interaction to block IFN activity. The possible outcome of PKR inhibition is not only IFN resistance and infection persistence but also cell growth promotion which ultimately leads to hepatocellular carcinoma (HCC).

Since E2 PePHD domain of HCV genotype 3a share less homology to PKR and eIF2 phosphorylation sites than do genotypes 1a and 1b. Therefore, it can be the most satisfactory reason that patients with 3a genotype react to interferon treatment more proficiently as compared to genotype 1a that shows maximum resistance to IFN therapy [37,38]. The relationship of E2-PePHD domain and its behavior during interferon therapy is quite indistinct. Some studies also explained the modifications happening in the PePHD domain during treatment. This work reveals that in non-responders, slight variations present in pretreated patients quickly evades during treatment [39]. HCV has developed ways to undo the antiviral effects of PKR [40,41] and it is assumed that IFN resistance may be due to phosphorylation of envelope proteins particularly of E2 protein. Our study focused on detecting such sites in our local isolate of HCV genotype 1a and confirmed two phosphorylation sites (S75 and S277) at the surface of E2 protein that interacted with CLK2 Kinase and YWHAZ. It is further proposed that this interaction might be responsible for HCV resistance to antiviral effects of IFN which could be confirmed by dephosphorylating these sites and analyzing its effects on PKR binding and inhibition.

## Conclusion

The role of envelope protein 2 (E2) of hepatitis C virus (HCV) is found to interact with double stranded RNA-dependent protein kinase (PKR) in previous studies. In the present study, the role of phosphorylation of E2 protein towards interferon sensitivity & PKR response was determined. HCV E2 protein was found to contain a sequence identical to phosphorylation sites of the interferon-inducible protein kinase (PKR). As a result E2 inhibits the kinase activity of PKR. This work will help to identify factors that can favor a successful innate immune response to HCV infection.

## Competing interests

The authors declare that they have no competing interests.

## Authors' contributions

MI conceived the study and critically reviewed the manuscript. SA performed, nucleotide sequencing and analyzed the results. SA, MA, AH and MI drafted the manuscript. MA, MA, MI, SB, SS, IR, LA and MS participated in data analysis. All the authors studied and approved the final manuscript.
